# Two Decades after Mandibuloacral Dysplasia Discovery: Additional Cases and Comprehensive View of Disease Characteristics

**DOI:** 10.3390/genes12101508

**Published:** 2021-09-26

**Authors:** Isabelle Jéru, Amira Nabil, Gehad El-Makkawy, Olivier Lascols, Corinne Vigouroux, Ebtesam Abdalla

**Affiliations:** 1 Inserm UMR_S938, Saint-Antoine Research Center, Institute of Cardiometabolism and Nutrition, Sorbonne University, 75012 Paris, France; olivier.lascols@aphp.fr (O.L.); corinne.vigouroux@aphp.fr (C.V.); 2 Department of Molecular Biology and Genetics, Assistance Publique-Hôpitaux de Paris, Saint-Antoine University Hospital, 75012 Paris, France; 3 Department of Human Genetics, Medical Research Institute, Alexandria University, Alexandria 21561, Egypt; amiranabil@alexu.edu.eg (A.N.); gehadelmakkawy@gmail.com (G.E.-M.); 4 National Reference Center for Rare Diseases of Insulin Secretion and Insulin Sensitivity (PRISIS), Department of Endocrinology, Assistance Publique-Hôpitaux de Paris, Saint-Antoine University Hospital, 75012 Paris, France

**Keywords:** mandibuloacral dysplasia, *LMNA*, genotype–phenotype correlation, lipodystrophy, acro-osteolysis

## Abstract

Pathogenic variants in the *LMNA* gene cause a group of heterogeneous genetic disorders, called laminopathies. In particular, homozygous or compound heterozygous variants in *LMNA* have been associated with “mandibuloacral dysplasia type A” (MADA), an autosomal recessive disorder, characterized by mandibular hypoplasia, growth retardation mainly postnatal, pigmentary skin changes, progressive osteolysis of the distal phalanges and/or clavicles, and partial lipodystrophy. The detailed characteristics of this multisystemic disease have yet to be specified due to its rarity and the limited number of cases described. Here, we report three unrelated Egyptian patients with variable severity of MAD features. Next-generation sequencing using a gene panel revealed a homozygous c.1580G>A-p.Arg527His missense variant in *LMNA* exon 9 in an affected individual with a typical MADA phenotype. Another homozygous c.1580G>T-p.Arg527Leu variant affecting the same amino acid was identified in two additional patients, who both presented with severe manifestations very early in life. We combined our observations together with data from all MADA cases reported in the literature to get a clearer picture of the phenotypic variability in this disease. This work raises the number of reported MADA families, argues for the presence of the founder effect in Egypt, and strengthens genotype–phenotype correlations.

## 1. Introduction

Mandibuloacral dysplasia (MAD) is a rare autosomal recessive progeroid disorder with clinical and genetic heterogeneity, characterized by growth retardation, craniofacial dysmorphism, clavicular resorption, acro-osteolysis, and skin abnormalities associated with lipodystrophy and insulin-resistance related metabolic complications. The first cases were clinically described by Young et al. in 1971 [1]. Two major types of MAD were differentiated according to body fat distribution patterns and were linked to different genetic defects: type A (MADA; OMIM #248370), characterized by a partial form of lipodystrophy, caused by pathogenic variants in the *LMNA* gene encoding lamin A/C [2]; and type B (MADB; OMIM #608612), presenting with generalized lipodystrophy, caused by molecular defects in the zinc metalloproteinase (*ZMPSTE24*) gene [3]. A third type of mandibuloacral dysplasia progeroid syndrome (MDPS; OMIM #619127) due to biallelic variants in the MTX2 gene encoding metaxin-2 (*MTX2*) was recently described [4].

Mutations in *LMNA* give rise to very diverse clinical phenotypes collectively called “primary laminopathies” that include skeletal myopathies such as Emery–Dreifuss muscular dystrophy and limb–girdle muscular dystrophy, premature aging syndromes such as Hutchinson–Gilford progeria syndrome (HGPS), restrictive dermopathy, and mandibulo acral dysplasia, as well as isolated cardiac disease with arrhythmia and dilated cardiomyopathy with conduction system disease [5]. Thus, MADA is a laminopathy caused by homozygous and compound heterozygous *LMNA* mutations [2,6,7]. Among the different pathogenic variants identified, the most frequent is the homozygous c.1580G>A variant in exon 9 of the *LMNA* gene, which changes Arginine 527 into histidine (p.Arg527His). Severe forms of MADA associated with progeroid features were also described in patients harboring different *LMNA* variants [8,9,10,11,12,13,14].

Lamins A/C, encoded by *LMNA*, are major components of the nuclear lamina and play a fundamental role in the maintenance of the size and shape of the nucleus and in several nuclear processes such as transcription, chromatin organization, and DNA replication [15]. The pathogenic variants identified in patients with MADA induce an accumulation of prelamin A in the nucleus [16,17,18] so that the first pathogenic event in MADA could be the toxic accumulation of unprocessed prelamin A [19]. Consequently, a marked alteration of the nuclear architecture and chromatin disorganization, which become more severe in older patients, are observed in cells from MADA patients [18]. Impaired preadipocyte differentiation leading to lipodystrophy could also result from the accumulation of prelamin A in these cells [17].

Although the first patients carrying *LMNA* variants were reported nearly 20 years ago, the number of reported cases remains limited, and most studies correspond to case reports. Here, we report three Egyptian patients with MADA and carrying homozygous variants in the *LMNA* gene. Based on these novel cases and a comprehensive review of the literature, this study provides a synthetic view of the clinical features and natural history of this very rare disorder.

## 2. Materials and Methods

### 2.1. Study Approval

The research was reviewed and approved by the Ethics Committee of the Medical Research Institute-Alexandria University (Alexandria, Egypt) and by the CPP Ile de France 5 Research Ethics Board (Paris, France). All study participants/legal guardians were asked to volunteer to the study and provided a signed informed consent (in concordance with the Declaration of Helsinki).

### 2.2. Screening of a Gene Panel by Next-Generation Sequencing

Peripheral blood samples were collected from all the probands and their parents. Genomic DNA was extracted from the peripheral blood samples by using the QIAamp DNA Blood Mini Kit (QIAGEN, Valencia, CA, USA). A panel of 23 genes involved in lipodystrophic syndromes was tested [20]. The list of genes in this panel is as follows: *LMNA*, *LMNB2*, *AGPAT2*, *BSCL2*, *PPARG*, *INSR*, *CAV1*, *PTRF*, *PLIN1*, *CIDEC*, *PIK3R1*, *PLD3*, *AKT2*, *LIPE*, *DYRK1B*, *NSMCE2*, *POC1A*, *LMF1*, *PCYT1A*, *POLD1*, *TBC1D4*, *PSMB8*, *ZMPSTE24*. Exons and flanking intronic sequences of these genes were captured from fragmented DNA with the SeqCapEZ enrichment protocol (Roche NimbleGen, Madison, WI, USA). Paired-end massively parallel sequencing was achieved on a MiSeq platform (Illumina, San Diego, CA, USA). Bioinformatic analysis was performed using the Sophia DDM pipeline^®^ (Sophia Genetics, Lausanne, Switzerland).

### 2.3. Sanger Sequencing

*LMNA* variants were confirmed by Sanger sequencing with the BigDye Terminator v3.1 sequencing kit (Thermo Fisher Scientific, Waltham, MA, USA) after polymerase chain reaction (PCR) amplification of exon 9 and flanking intronic sequences. Data were analyzed on a 3500xL Dx device with the SeqScape v2.7 software (Thermo Fisher Scientific, Waltham, MA, USA). *LMNA* variants were described based on the longest isoform (NM_170707.2) using Alamut 2.11 (Sophia Genetics, Lausanne, Switzerland) and Human Genome Variation Society guidelines.

## 3. Case Reports

### 3.1. Proband 1

**Figure 1:**
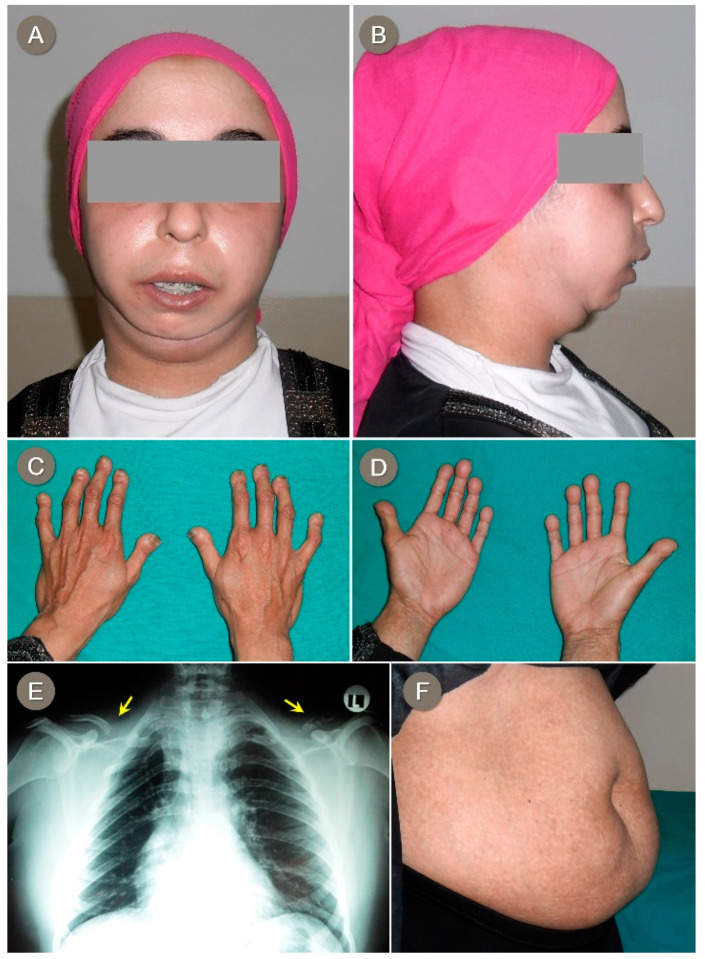
Proband 1 shows an oval-shaped, bird-like face; wide prominent eyes; full cheeks; submental obesity; arched, heavy eyebrows; a narrow, prominent nasal bridge and shallow philtrum (**A**); a beaked nose and severe mandibular hypoplasia (**B**); bulbous fingertips and nail dystrophy (**C**,**D**); marked bilateral clavicular hypoplasia evident on chest X-ray (**E**); and marked abdominal obesity and reticular/mottled hyperpigmentation (**F**).

A 27-year-old woman, born to Egyptian consanguineous healthy parents, was referred for genetic assessment for her progressive condition of mandibular hypoplasia (Figure 1A,B), lipodystrophy of extremities, loss of scalp hair, and skin hyperpigmentation. The condition started at around the age of 14 years. The patient was born at term after an uneventful pregnancy, with normal growth parameters. All through her childhood, she had average physical growth, normal psychomotor development, and good scholastic performance. Notably, the patient underwent a number of unsuccessful plastic surgeries in an attempt to reverse the mandibular changes.

A thorough clinical examination revealed thin and sparse scalp hair; an oval-shaped, bird-like face; scleroderma-like facial skin; wide, prominent eyes; full cheeks; submental obesity; arched, heavy eyebrows; a narrow, prominent nasal bridge; a beaked nose; a shallow, long philtrum; an open mouth with overlapping upper incisors and dental crowding; and severe mandibular hypoplasia. Increased adipose tissue around the neck and marked abdominal obesity with reticular/mottled hyperpigmentation of the overlying skin were also noticed (Figure 1F). Moreover, skin thinning was noted over acral regions, with prominent veins due to the lack of subcutaneous fat. She also had broad, round-tipped terminal phalanges of the fingers and toes, giving a drumstick appearance, and broad, short dystrophic nails (Figure 1C,D). Plain chest X-ray showed marked hypoplasia of the right clavicle and severe osteolysis of the left (Figure 1E). Anthropometric measures revealed low weight (50 kg) and short stature (147 cm), with normal head circumference.

Next-generation sequencing (NGS), using a panel of 23 genes involved in lipodystrophic syndromes, was performed in a DNA sample from Patient 1. This analysis revealed a homozygous missense variant in exon 9 of the *LMNA* gene, c.1580G>A (NM_170707.2), affecting Arginine 527 (p.Arg527His). This genotype was confirmed by Sanger sequencing. According to the American College of Medical Genetics and Genomics (ACMG) criteria [21], this variant can be classified as “pathogenic”. Indeed, based on the gnomAD database, which reports variants from the general population, it is a very rare variant present at a frequency of 4 × 10^−5^ and has never been found in the homozygous state in healthy individuals. Multiple lines of computational evidence (SIFT, MutationTaster, REVEL, CADD) support a deleterious effect for this variant. A previous study reported that this variant causes accumulation of the lamin A precursor protein, a marked alteration of the nuclear architecture and, hence, chromatin disorganization [16]. In addition, this genotype was previously implicated in MADA [2,22,23,24], confirming the diagnosis in this patient.

### 3.2. Proband 2

The patient was a 5-year-old boy, born to Egyptian consanguineous healthy parents. He was born full term, with normal growth parameters, after an uneventful pregnancy. Since the age of 2 years, the parents noticed progressive swelling and stiffness of the fingers with changes in facial features. Motor, speech, and cognitive development were all normal.

**Figure 2:**
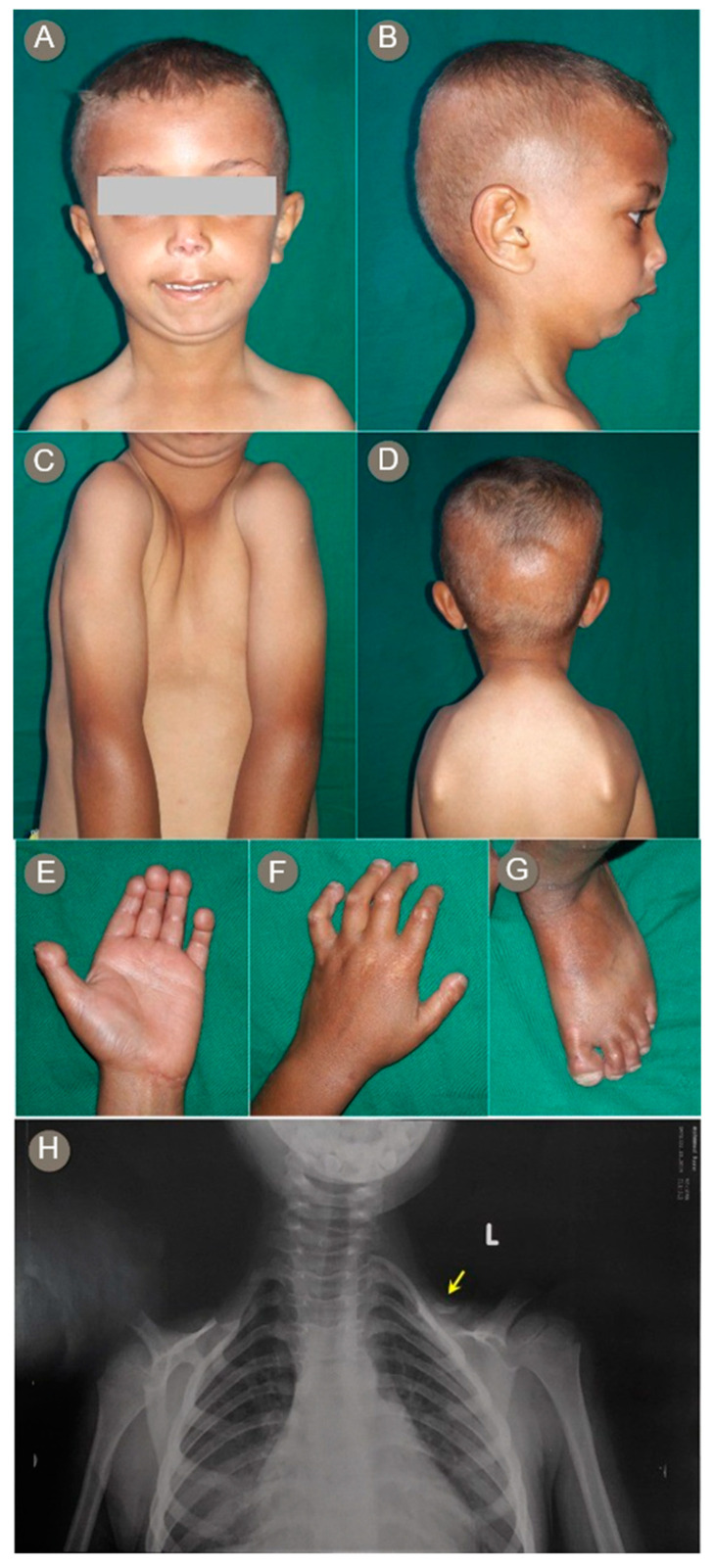
Proband 2 showing an oval face; wide eyes with periocular hyperpigmentation; arched eyebrows; a short, thin, upturned nose; a small mouth with limited opening (**A**); submental obesity and microretrognathia (**B**); narrow, sloping shoulders and prominent scapular wings (**C**); abnormal facility in opposing shoulders (**D**); short fingers with drumstick-shaped distal phalanges (**E**); flexed fingers with atrophic shiny overlying skin; hypopigmentation over the knuckles and broad, short, dystrophic nails (**F**); and round toe tips, nail dystrophy, and visible veins (**G**). Chest radiograph demonstrating severely hypoplastic/absent clavicles bilaterally (**H**).

On clinical examination, the patient showed loss of subcutaneous fat in the limbs and trunk and sclerodermatous changes over the face and extremities. We also noticed diffuse loss of scalp hair; an oval face; submental obesity; wide eyes; periocular hyperpigmentation; arched eyebrows; a short, thin, upturned nose; a small mouth with limited opening; and mandibular hypoplasia and microretrognathia (Figure 2A–C). The fingers were markedly short with stiff interphalangeal joints that resulted in a flexion deformity and decreased mobility of the fingers; drumstick-shaped distal phalanges; and broad, short nails (Figure 2E,F). The overlying skin appeared stretched, thin, atrophic, and shiny, with some rigidity and hypopigmentation over the knuckles. The toes also had round tips and broad, short nails (Figure 2G). The patient had a bell-shaped chest; narrow, sloping shoulders; prominent scapular wings; and abnormal facility in the opposing shoulders due to the clavicular hypoplasia (Figure 2C,D). Chest radiograph revealed a bilateral severely hypoplastic clavicle (Figure 2F). As in Proband 1, anthropometric measures revealed low weight (12.5 kg) and height (95 cm) for age, with normal head circumference (49.5 cm).

Screening of the gene panel revealed a homozygous missense variant in exon 9 of *LMNA* c.1580G>T (NM_170707.2); p.Arg527leu. The same variant was present in the parents of the proband in the heterozygous state, and the genotype was confirmed by Sanger sequencing. According to the ACMG criteria, this variant can be classified as “pathogenic”. This variant was absent from the gnomAD database. Multiple lines of computational evidence (SIFT, MutationTaster, REVEL, CADD) support its deleterious effect. Homozygosity for this variant was previously shown to be responsible for MADA [13,14], confirming the diagnosis in this patient.

### 3.3. Proband 3

A 2-year-old boy was born to Egyptian first cousins at term with a birth weight of 4.5 kg. Normal developmental history was confirmed by the parents. The parents gave a history of persistent fever at the age of 1 year, which lasted for 2 months, accompanied by chronic diarrhea. Afterward, the parents reported a progressive loss of scalp hair and recurrent finger swelling.

**Figure 3:**
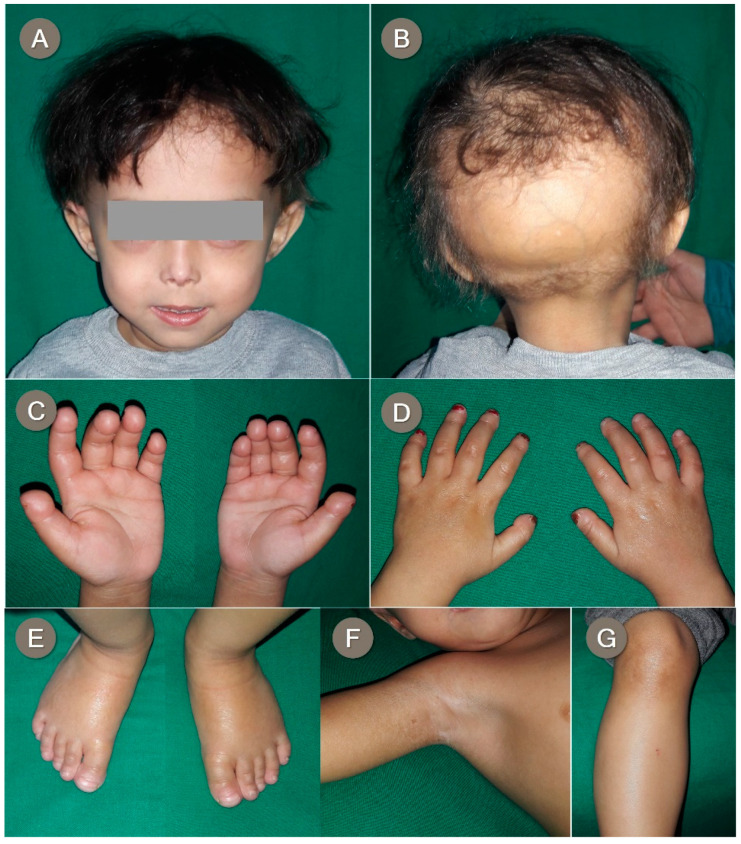
Proband 3 shows a receding anterior hairline; scanty scalp hair; bulbous cheeks with visible veins; wide eyes with periocular hyperpigmentation; a pointed nose; a small mouth and micrognathia (**A**); occipital alopecia and prominent scalp veins (**B**); round-tipped terminal phalanges of fingers (**C**); atrophic shiny skin on the dorsum of hands and broad, short, dystrophic nails (**D**); mild rounding of toe tips and broad, short nails (**E**); hyperpigmented thin skin over knees (**F**); and hyper- and hypopigmented patches on axilla (**G**).

On physical examination, we noticed a receding anterior hairline, scanty scalp hair, total occipital alopecia, and prominent scalp veins (Figure 3B). In addition, the patient had an oval face, bulbous cheeks with visible veins, wide eyes, periocular hyperpigmentation, a tapered nasal tip, a small mouth, dental crowding, mandibular hypoplasia, and microretrognathia (Figure 3A). He also presented with round-tipped terminal phalanges of the fingers and toes with broad, hypoplastic nails (Figure 3C–E). In addition, hard shiny skin on the dorsum of the hands with subcutaneous nodules on the knuckles, pigmentary changes on the knees, and hypopigmented patches on the axilla were noted (Figure 3F,G). Similar to the other two probands, both weight and height were lower than the third centiles, while the head circumference was within the normal range for his age (weight: 9.5 kg, height: 78 cm, head circumference: 49 cm). On reviewing the chest radiograph, there was no evident clavicular osteolysis or dysplasia, only mild thinning bilaterally.

NGS panel testing and Sanger sequencing revealed that Patient 3 had the same genotype as Patient 2, since he carries the c.1580G>T (p.Arg527Leu) *LMNA* variant in the homozygous state, each mutated allele being inherited from one of the two parents.

The clinical characteristics of the three patients are summarized in Table 1. For Proband 1, the recorded clinical features were reminiscent of a classical phenotype of MADA. However, the manifestations and early presentation in Probands 2 and 3 suggested a more severe form of laminopathy with progeroid features. Notably, apart from the *LMNA* variants, no other molecular defect was identified in the three probands in the 23 genes of the panel, which comprises ZMPSTE24 (see Section 2 for the complete list of genes).

Figure S1 (Supplementary Materials) illustrates the Sanger sequencing electropherograms of exon 9 of *LMNA*, which revealed a homozygous c.1580 G>A mutation in P1 and a homozygous c.1580G>T mutation in P2 and P3.

### 3.4. Comprehensive Review of the Literature

Based on a systematic review of the literature, we aimed to better delineate the main characteristics and natural history of the disease. We identified 40 patients affected with MAD from 16 reports. Table 2 summarizes the pathogenetic variants identified so far in *LMNA* and the main clinical features of the published cases, in relation to those of the three patients investigated herein. The male to female sex ratio was 20/23, consistent with the autosomal recessive disease inheritance mode. The age at clinical investigation in MADA was usually between the first and second decade of life, with an average age at investigation of 11 years. However, the age at onset of symptoms is usually earlier due to delay in diagnosis. We observed that the clinical presentation of MADA is quite homogeneous. Nevertheless, it is characterized by a complex clinical picture, with the involvement of multiple organ systems to differing extents in different individuals. We propose a classification of the major and minor clinical features of MADA, according to their frequency in the reported cases. The major clinical features for the diagnosis of MADA, present in more than 75% of patients include acro-osteolysis (100%), lipodystrophy (98%), mandibular hypoplasia (95%), clavicular hypoplasia (93%), growth retardation (79%), and a beaked nose (77%). Manifestations present in 50–75% of patients comprise mottled skin pigmentation (72%), prominent cheeks (70%), prominent eyes (65%), and dental crowding 63%). Alopecia is a minor disease sign reported in only half of the patients so far.

## 4. Discussion

Mandibuloacral dysplasia type A is a rare autosomal recessive disorder with multisystemic manifestations involving skin, skeleton, and adipose tissue. Its complex presentation leads to delay in diagnosis and justifies careful clinical evaluation and multidisciplinary care. The current study provides additional data on this rare disorder and a complete overview of clinical features and disease characteristics.

The first signs of MADA occur usually in early childhood and become more evident in the second decade of life [19,29]. MADA is also characterized by lipodystrophy pattern type A, described as loss of subcutaneous fat in the extremities and normal or heightened presence of fatty tissue in the neck and trunk [2]. The three Egyptian patients investigated herein displayed variable severity of MADA. Proband 1 exhibited all constant signs of MADA, and these features started to be noticed around the age of 14 years. The other two patients (P2 and P3), however, had disease onset in infancy and shared the same dysmorphic features, besides progressive loss of scalp hair, leading to alopecia, and a more extensive form of lipodystrophy, which only spared the face and neck. Thus, the clinical diagnosis of an atypical form of progeroid laminopathy or of MADB was more likely. Indeed, more pronounced accelerated aging features have been reported in MADB [3].

In this study, we identified two known disease-causing homozygous pathogenic variants in *LMNA* exon 9. The most common genetic cause of MADA, c.1580G>A (p.Arg527His), was found in Proband 1, while a variant affecting the same protein residue, c.1580G>T (p.Arg527Leu), was detected in Probands 2 and 3. Based on the review of the literature, we observed that the other identified *LMNA* pathogenic variants, albeit less frequent, show clustering in the C-terminal globular domain common to prelamin A and lamin C, suggesting a common pathophysiological mechanism for all variants [2,22,23,24].

We took a particular interest in the less frequent p.Arg527Leu pathogenic variant. In 2012, two research groups independently identified this variant in the homozygous state in Egyptian patients with atypical MADA and progeroid features [13,14]. On exhaustive literature searching, these two studies were the only literature reports related to this *LMNA* variant. According to Al-Haggar et al., the replacement of arginine at position 527 of the wild-type lamin A by a neutral, hydrophobic leucine is predicted to completely destroy the salt bridge, which arginine forms with the glutamate at position 537 to stabilize the structure of the conserved C-terminal immunoglobulin-like domain of this protein [30,31]. Thus, it results in the destabilization of the immunoglobulin-like domain structure and increases the liability for protein aggregation [13]. Such an alteration most probably affects the interactions of lamin A with other proteins, thus influencing multiple cellular processes. In contrast, Al-Haggar et al. showed that the replacement of Arginine 527 by a basic histidine only results in the destabilization of the salt-bridge formation with less severe consequences [13]. This p.Arg527Leu variant was previously shown to be present at high frequency in Egypt. On performing restriction fragment-length polymorphism analysis for 178 unrelated individuals, Al-Haggar et al. showed that up to 1.12% of inhabitants of Northeast Egypt might be heterozygous carriers of this mutation [13]. A previous report of the same *LMNA* p.Arg527Leu substitution mutation by Amr et al. 2012 [14], in three seemingly unrelated Egyptian families, and its detection in our cohort are consistent with a founder effect of this variant in this region, especially since the three families included in this study originate from the Northwestern region of Egypt.

In Patients 2 and 3, the p.Arg527Leu variant was associated with a severe form of MADA, characterized by early onset and the presence of a full set of typical MADA symptoms, together with some progeroid features. These observations further emphasize the previously proposed genotype–phenotype correlation between this variant and more pronounced accelerated aging. Notably, most previous research highlighted mild accelerated aging in MADA cases compared to patients with MADB and other progeroid syndromes [18,22,23,24]. Analysis of additional patients, particularly with the use of modern sequencing technologies, will certainly further increase our knowledge on genotype–phenotype correlations and reveal whether additional genes may also be involved in modulating the phenotype of MADA and related laminopathies.

## Figures and Tables

**Table 1:** Clinical characteristics of the three patients with MADA investigated herein.

Clinical Characteristics	Proband 1	Proband 2	Proband 3
Age	27 years	5 years	2 years
Sex	Female	male	male
Age of onset	14 years	2 years	1 year
Height for age	<P3	<P3	<P3
Weight for age	<P3	<P3	<P3
Microcephaly	-	-	-
Prominent eyes	+	-	-
Full cheeks	+	-	+
Micrognathia	+++	++	+
Retrognathia	+++	++	+
Dental crowding	++	-	+
Beaked/Pinched/pointed nose	+	+	+
Alopecia/Sparse hair	+	++	+++
Abdominal obesity	+++	-	-
Submental obesity	++	+	-
Clavicle hypoplasia	++	+++	+
Lipodystrophy of extremities	+	+	-
Acroosteolysis	++	++	+
Finger rounding (drumstick shaped distal phalanges)	+++	++	+
Joint contractures	+	++	-
Mottling/Hyperpigmentation	++	-	+

-: absent, +: mild, ++: moderate, +++: severe.

**Table 2:** Full list of published cases of MADA with biallelic *LMNA* pathogenic variants (in chronological order) and their corresponding phenotypes. For each gene mutation, the nucleotide mutation (c.) and corresponding amino acid change (p.) are indicated. N/A: not available.

References	Pathogenic Variant	Genotype	Phenotype					Phenotype									
			Typical or Atypical	Number of Cases Reported	Gender	Age at Investigation	Growth Retardation/Short Stature	Prominent Eyes	Beaked/Pointed Nose	Prominent Cheeks	Dental Crowding	Mandibular Hypoplasia	Clavicular Hypoplasia/Osteolysis	Acro-osteolysis	Lipodystrophy (A/B)	Mottled Skin Pigmentation	Alopecia
Novelli et al., 2002 [2].	c.1580 G>A-p.(Arg527His)	Homozygous	Typical MADA	9	4 F 5 M	1 case: 35 years 8 cases: N/A	9/9	N/A	1/9 8/9 N/A	1/9 8/9 N/A	N/A	9/9	9/9	9/9	9/9 A	9/9	5/9
Cao and Hegele, 2003 [8].	c.1580 G>T-p.(Arg471Cys)/ c.1623 C>T-p. (Arg527Leu)	Compound heterozygous	Atypical MADA with progeroid features	1	F	28 years	N/A	N/A	N/A	N/A	N/A	1/1	1/1	1/1	1/1 A	N/A	1/1
Simha et al., 2003 [22].	c.1580 G>A-p.(Arg527His)	Homozygous	Typical MADA	2	2 F	20 years 16 years	2/2	2/2	2/2	2/2	2/2	2/2	2/2	2/2	2/2 A	2/2	0/2
Shen et al., 2003 [23].	c.1580 G>A-p.(Arg527His)	Homozygous	Typical MADA	1	M	12 years	0/1	1/1	1/1	1/1	1/1	1/1	1/1	1/1	1/1 A	1/1	0/1
Plasilova et al., 2004 [9].	c.1626 G>C-p.(Lys542Asn)	Homozygous	Typical MADA	4	2 F 2 M	4.5 years 10 years 15 years 17 years	4/4	4/4	4/4	4/4	3/4	4/4	4/4	4/4	4/4 B	4/4	4/4
Verstraeten et al., 2006 [10].	c.1583 C>T-p.(Thr528Met)/ c.1619 T>C-p.(Met540Thr)	Compound heterozygous	Atypical MADA with progeroid features	1	M	18–24 months	1/1	1/1	1/1	1/1	1/1	1/1	1/1	1/1	1/1 A	0/1	1/1
Kosho et al., 2007 [25].	c.1585 G>A-p.(Ala529Thr)	Homozygous	Typical MADA	1	F	mid-20s	1/1	1/1	1/1	1/1	0/1	1/1	1/1	1/1	1/1 A	1/1	1/1
Lombardi et al., 2007 [7].	c.1580 G> A-p.(Arg527His)/ c.1318 G> A-p.(Val440Met)	Compound heterozygous	Typical MADA with skeletal muscle involvement	1	F	22 years	0/1	N/A	1/1	N/A	0/1	0/1	0/1	1/1	N/A	0/1	0/1
Agarwal et al., 2008 [12].	c.1579 C>T-p.(Arg527Cys)	Homozygous		1	F	7 years	1/1	N/A	1/1	1/1	1/1	1/1	1/1	1/1	1/1	1/1	1/1
Zirn et al., 2008 [11].	c.1623 C>T-p.(Arg471Cys)	Homozygous	Typical MADA	1	F	3 years	0/1	1/1	1/1	1/1	1/1	1/1	1/1	1/1	1/1 A	0/1	0/1
Garavelli et al., 2009 [24].	c.1580 G>A-p.(Arg527His)/ c.1318 G>A-p.(Val440Met)	Homozygous		2	1 F 1 M	4 years 5 years	0/2	2/2	2/2	2/2	1/2	1/2	2/2	2/2	2/2	1/2	N/A
Amr, Mostafa, and El-Kamah, 2012 [14].	c.1580 G>T-p.(Arg527Leu)/ c.1579 C>T-p.(Arg527Cys)	Homozygous	MADA with lipodystrophy and progeroid features	4	2 F 2 M	9 years 18 years 7 years 8 years	4/4	4/4	4/4	4/4	4/4	4/4	4/4	4/4	4/4 A	N/A	N/A
Al-Haggar et al., 2012 [13].	c.1580 G>T-p.(Arg527Leu)	Homozygous	Atypical MADA with progeroid features	3	3 F	2.5 years 5 years 3 years	3/3	3/3	3/3	3/3	3/3	3/3	3/3	3/3	3/3 A	2/3	2/3
Luo et al., 2014 [26].	c.1579 C>T-p.(Arg527Cys)	Homozygous	Typical MADA	3	2 F 1 M	10 months 12 months 8 months	3/3	2/3	2/3	2/3	2/3	3/3	2/3	3/3	3/3 B	3/3	3/3
Yassaee et al., 2016 [27].	c.1620 G>A-p.(Met540Ile)	Homozygous	Typical MADA	1	M	10 months	1/1	1/1	1/1	1/1	1/1	1/1	1/1	1/1	1/1 B	N/A	N/A
Sakka et al., 2021 [28].	c.1580 G>A-p.(Arg527His)	Homozygous	Typical MADA with growth hormone deficiency and cardiomyopathy	5	1 F 4 M	12 years 11 years 3 years 9 years 48 years	4/5	5/5	5/5	4/5	5/5	5/5	4/5	5/5	5/5	5/5 A	1/5
Current study	P1: c.1580 G>A-p.(Arg527His) P2/P3: c.1580 G>T-p.(Arg527Leu)	Homozygous		3	1 F 2 M	27 years 5 years 2 years	3/3	1/3	3/3	2/3	2/3	3/3	3/3	3/3	3/3	2/3	3/3
Total				43	23 F 20 M		34/43	28/43	33/43	30/43	27/43	41/43	40/43	43/43	42/43	31/43	22/43

## Data Availability

Not applicable.

## Supplementary Materials

The following are available online at https://www.mdpi.com/article/10.3390/genes12101508/s1, Figure S1: the Sanger sequencing electrophero-grams of exon 9 of *LMNA*, which revealed a homozygous c.1580 G>A mutation in P1 and a homozygous c.1580G>T mutation in P2 and P3.
