# Supplementary material for: Two Decades after Mandibuloacral Dysplasia Discovery: Additional Cases and Comprehensive View of Disease Characteristics
Source: Genes (Basel). 2021 Sep 26;12(10):1508. doi: 10.3390/genes12101508 (PMC8535562; doi:10.3390/genes12101508)
Supplement: Supplementary file 1 [file genes-12-01508-s001.zip › genes-1381760-supplementary.pdf]

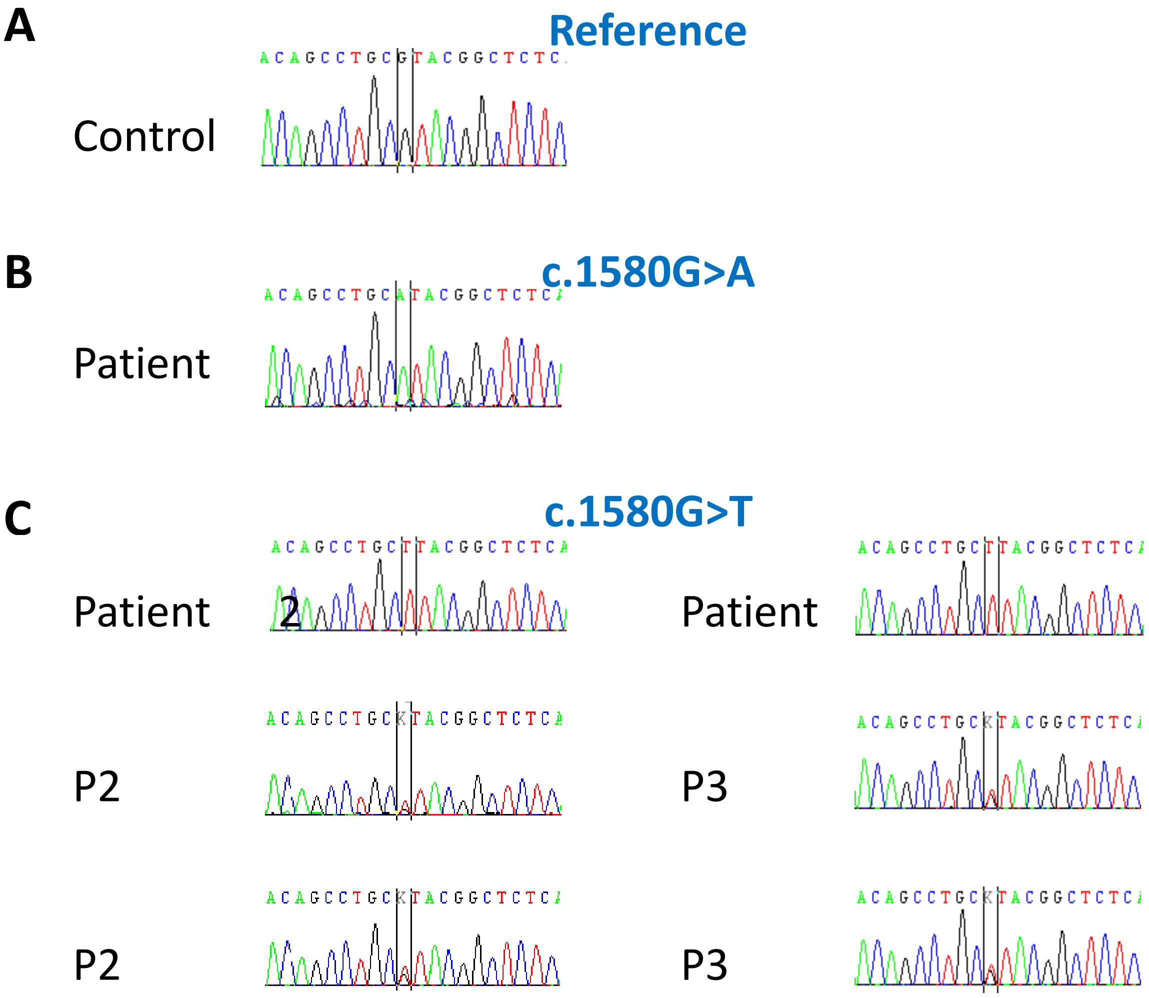

Figure S1. The Sanger sequencing electrophero-grams of exon 9 of LMNA, which revealed a homozygous c.1580 G>A mutation in P1 and a homozygous c.1580G>T mutation in P2 and P3.
